# circ_0030018 promotes glioma proliferation and metastasis

**DOI:** 10.1515/tnsci-2020-0175

**Published:** 2021-06-09

**Authors:** Yun Shao, Zhengxiang Yang, Weifeng Miao, Xiangrong Yu, Yiping Wu, Yi Pu

**Affiliations:** Department of Neurosurgery, The Affiliated Wuxi People’s Hospital of Nanjing Medical University, No. 299 Qingyang Road, Wuxi City, Jiangsu, 214023, China

**Keywords:** glioma, circ_0030018, miR-194-5p, TRIM44

## Abstract

**Background:**

Circular RNA (circRNA) plays an essential role in tumor progression, including glioma. circ_0030018 is a newly discovered circRNA that is highly expressed in glioma. However, its role and mechanism in glioma need to be further elucidated.

**Methods:**

The expression of circ_0030018, microRNA (miR)-194-5p, and *tripartite motif containing 44 (TRIM44)* was examined using quantitative real-time PCR. Cell proliferation, migration, invasion, and apoptosis were determined using MTT assay, colony formation assay, transwell assay, and flow cytometry. Moreover, dual-luciferase reporter assay and RNA pull-down assay were used to verify the interactions among circ_0030018, miR-194-5p, and *TRIM44*. The protein expression of TRIM44 was assessed by western blot analysis. Animal experiments were conducted to explore the role of circ_0030018 in glioma tumor growth *in vivo*.

**Results:**

circ_0030018 was overexpressed in glioma tissues and cells, and its silencing could inhibit glioma cell proliferation, migration, invasion, and accelerate apoptosis. miR-194-5p could be sponged by circ_0030018, and its overexpression could hinder the progression of glioma cells. Further experiments revealed that miR-194-5p inhibitor reversed the negative regulation of circ_0030018 knockdown on glioma cell progression. In addition, TRIM44 was a target of miR-194-5p, and its downregulation could repress glioma cell progression. Overexpressed TRIM44 reversed the inhibition effect of miR-194-5p on glioma cell progression. Animal experiments suggested that circ_0030018 knockdown could reduce glioma tumor growth through regulating miR-194-5p and TRIM44.

**Conclusion:**

Our 8data showed that circ_0030018 enhanced glioma progression by sponging miR-194-5p to regulate TRIM44, indicating that circ_0030018 might be a potential treatment target for glioma.

## 1 Introduction

Glioma is the common primary central nervous system malignant tumor in the skull, which is formed by the abnormal proliferation of glial cells [1,2]. Due to the characteristics of invasive growth, the treatment of glioma is very difficult and the postoperative recurrence rate of patients is very high [3,4]. In recent years, targeted therapy has been proposed to improve the survival of patients with glioma [5,6]. Therefore, elucidating the targets affecting the progress of glioma may bring more new ideas for the targeted therapy of glioma.

Circular RNA (circRNA) is a kind of noncoding RNA formed by reverse-splicing [7,8]. circRNA has strong stability due to its special circular structure, so it has great application prospects as a therapeutic target for diseases [9,10]. In a variety of tumor types, circRNA has been discovered to be abnormally expressed and is associated with the biological behaviors of tumor cells, including glioma [11,12]. circ_0030018 is located at chr13 with 2,656 bp. According to the transcript NM_006475.3, circ_0030018 is located in the second half of *POSTN* gene starting from 649 bp and contains all subsequent exons. In past studies, Wang et al. used high-throughput sequencing to find that circ_0030018 was significantly overexpressed in glioblastoma [13]. Later, Yang et al. proposed that the high circ_0030018 expression could enhance glioma cell growth and metastasis [14]. However, the current research on circ_0030018 in glioma is still in the preliminary stage, and more studies are needed to confirm whether it can be used as a therapeutic target for glioma.

Most circRNA can be used as the sponge of microRNA (miRNA) to regulate gene expression, which is a key way to clarify the molecular mechanism of circRNA [15,16]. In addition to exploring the role of circ_0030018 in glioma, we also proposed the hypothesis of the circRNA/miRNA/mRNA axis to reveal its mechanism. Using bioinformatics prediction, we found that circ_0030018 and miR-194-5p had targeted binding sites. Besides, we also confirmed that *tripartite motif containing 44 (TRIM44)* was a target of miR-194-5p, and its expression was indirectly regulated by circ_0030018. Our study mainly explored the role and mechanism of circ_0030018 in glioma to provide new evidence for circ_0030018 to be a therapeutic target for glioma.

## 2 Materials and methods

### 2.1 Tissue samples

Thirty five cases of glioma tissues (from glioma patients) and 20 cases of normal brain tissues (from volunteer donors or patients undergoing intracerebral trauma surgery) were collected from the Affiliated Wuxi People’s Hospital of Nanjing Medical University. All tissues were immediately preserved in liquid nitrogen after surgical removal.


**Informed consent:** All patients or family members had signed informed consents.

### 2.2 Cell culture and transfection

Human glioma cell lines (LN229, SHG44, U251, and T98G) and normal glial cell line (HEB) were obtained from Biovector National Typical Culture Center (NTCC, Beijing, China). Glioma cell lines were cultured in DMEM medium and HEB cells were grown in RPMI-1640 medium (all from Gibco, Grand Island, NJ, USA). Both mediums were supplemented with 10% fetal bovine serum (FBS; Gibco) and 1% Penicillin-Streptomycin Solution (Sangon Biotech, Shanghai, China). All cells were cultured in an incubator at 37°C with 5% CO_2_.

For cell transfection, all oligonucleotides and vectors were synthesized by Ribobio (Guangzhou, China), including the small interference RNA (siRNA) for circ_0030018 (si-circ_0030018: 5′-GAATGAAATTAGTTGTCACTG-3′) and *TRIM44* (si-*TRIM44*: 5′-AAUAGGUACUCAAAUCAAGCC-3′), or circ_0030018 lentiviral short hairpin RNA (sh-circ_0030018: 5′-CCGGAAATTAGTTGTCACTGTTAATCTCGAGATTAACAGTGACAACTAATTTTTTTTG-3′), miR-194-5p mimic or inhibitor (miR-194-5p or in-miR-194-5p), the pcDNA overexpression vector of *TRIM44*, and their controls (si-circ-NC, si-NC, sh-NC, miR-NC, in-miR-NC, or vector). Lipofectamine 3000 Reagent (Invitrogen, Carlsbad, CA, USA) was used to transfect them into LN229 and U251 cells.

### 2.3 Quantitative real-time PCR (qRT-PCR)

Trizol reagent (Invitrogen) was used for RNA extraction. Afterwards, the RNA was reverse-transcribed into cDNA with High Capacity cDNA Reverse Transcription Kit (ABI, Foster City, CA, USA). Finally, PCR was performed using SYBR Green PCR Master Mix (ABI). For data analysis, β-actin or U6 was used as the endogenous control, and data were calculated using 2^−ΔΔCt^ method [17]. The sequence primers were listed as below: circ_0030018, F 5′-GTTTGGACTTGGGAACAGGA-3′, R 5′-CACCATTTGTTGCAATCTGG-3′; *POSTN*, F 5′-CTCATAGTCGTATCAGGGGTCG-3′, R 5′-ACACAGTCGTTTTCTGTCCAC-3′; miR-194-5p, F 5′-GCCGAGTGTAACAGCAACTCCA-3′, R 5′-GCAGCTCAGTAACAGTCCGC-3′; *TRIM4*4, F 5′-CCATCTGGCCGAATACGTCC-3′, R 5′-TGCCTCGCTTTCTATCTCCCT-3′; β-actin, F 5′-ATAGCACAGCCTGGATAGCAACGTAC-3′, R 5′-CACCTTCTACAATGAGCTGCGTGTG-3′; U6, F 5′-CTCGCTTCGGCAGCACA-3′, R 5′-AACGCTTCACGAATTTGCGT-3′. The forward primers of circ_0030018 were 2,581 to 2,601 bp, and the reverse primers were the reverse sequence of 38 to 58 bp. The PCR amplification sequence for circ_0030018 was 133 bp. The forward primers of *POSTN* were 131 to 152 bp, and the reverse primers were the reverse sequence of 248 to 268 bp. The PCR amplification sequence for *POSTN* was 138 bp.

### 2.4 RNase R assay

As previously described [18], the RNA (1 μg) extracted from LN229 and U251 cells was incubated with 2 U RNase R (Epicentre, Madison, WI, USA) for 30 min at 37°C. The incubated RNA was then used to perform qRT-PCR to detect circ_0030018 and *POSTN* expression. Relative expression is the normalization of the expression of relevant genes in the mock (the RNA non-treated with RNase R).

### 2.5 Cell viability analysis

LN229 and U251 cells were collected and then seeded into 96-well plates. After the cells attached to the plates, 20 μL 0.5% MTT solution (Solarbio, Beijing, China) was added into cells at 0, 24, 48, or 72 h, respectively. After 4 h, the medium was removed and 150 μL DMSO (Solarbio) was added into cells for shaking 10 min. At 490 nm, the optical density (OD) value was detected by microplate reader to assess relative cell viability as previously described [18].

### 2.6 Colony formation assay

As previously described [19], LN229 and U251 cells were harvested and then plated into 6-well plates (150 cells). The cells were cultured at 37°C for 14 days. After that, the cell medium was removed and the forming colonies were fixed in paraformaldehyde and dyed using crystal violet (Beyotime, Shanghai, China). The colonies were photographed and counted by microscope.

### 2.7 Cell migration and invasion analysis

As previously described [14], Transwell chambers (Corning Incorporate, Corning, NY, USA) pre-coated with Matrigel (Corning Incorporate) were used for detecting cell invasion, while non-coated ones were used for measuring cell migration. LN229 and U251 cells were seeded to the upper chamber with DMEM medium. The medium supplemented with 10% FBS was added into the lower chamber. After 24 h, the cells migrated and invaded into the lower chamber were fixed in paraformaldehyde and dyed with crystal violet. Under microscope, the cells were photographed (100×) and its numbers were counted.

### 2.8 Cell apoptosis analysis

Annexin V-FITC/propidine iodide (PI) Apoptosis Kit (Biovision, Milpitas, CA, USA) was used to assess cell apoptosis. In brief, LN229 and U251 cells were harvested and suspended with 1× Binding Buffer. After staining with Annexin V-FITC and PI, the apoptosis rate of cells was analyzed by FACScan flow cytometer as previously described [18].

### 2.9 Dual-luciferase reporter assay

The sequences of circ_0030018 or *TRIM44* 3′UTR containing the predicted binding sites or matched mutated sites of miR-194-5p were cloned into pmirGLO vectors (Promega, Madison, WI, USA), named as the wild-type (wt) or mutate-type (mut) vector, respectively. Lipofectamine 3000 Reagent was used to transfect with the vectors and miR-194-5p mimic or miR-NC into LN229 and U251 cells. After 48 h, luciferase activity was analyzed by Dual-Luciferase Reporter Gene Assay Kit (Beyotime) as previously described [14].

### 2.10 RNA pull-down assay

RNA pull-down assay was performed as previously described [20]. The bio-miR-194-5p probe and its control (bio-NC) probe were synthesized by Sangon Biotech. LN229 and U251 cells were transfected with bio-miR-194-5p probe and bio-NC probe. Forty eight hours later, the cells were lysed and the cell lysates were co-cultured with streptavidin magnetic beads (Roche) at 4°C overnight. Then, RNA complexes were purified for detecting the expression of circ_0030018 and TRIM44 using qRT-PCR.

### 2.11 Western blot (WB) analysis

As previously described [21], LN229 and U251 cells were harvested and lysed by RIPA lysis buffer (Beyotime). The protein was quantified by BCA Protein Assay Kit, and then 20 μg protein samples were electrophoresed by SDS-PAGE gel followed by transferring onto a PVDF membrane (Roche, Basel, Switzerland). After that, the membrane was incubated with nonfat milk for 2 h and then hatched with specific primary antibodies targeting TRIM44 (1:2,000, Abcam, Cambridge, MA, USA), POSTN (1:1,000, Abcam), or β-actin (1:1,000, Abcam) at 4°C overnight. After the membrane was cultured with Goat anti-Mouse or Rabbit IgG (1:20,000, Abcam) for 1 h, ECL Western Blotting Substrate Kit (Biovision) was used to visualize the protein bands in the membrane

### 2.12 Animal experiments

Animal experiments were performed as previously described [18]. BALB/c male nude mice (*n* = 20, Vital River, Beijing, China) were randomly divided into four groups (*n* = 5 per group). LN229 and U251 cells were transfected with sh-NC or sh-circ_0030018 for 48 h. After that, the cells were collected and resuspended with PBS (2 × 10^6^/0.2 mL PBS), and then subcutaneously injected into the nude mice. Every week, tumor length and width were detected for calculating tumor volume. Four weeks later, the mice were euthanized and the tumor tissues were obtained. The tumor in each group was photographed, and then the expression of circ_0030018, miR-194-5p, and TRIM44 was detected.


**Ethical approval:** The research related to human use has been complied with all the relevant national regulations, institutional policies and in accordance with the tenets of the Helsinki Declaration, and has been approved by the authors’ institutional review board at Affiliated Wuxi People’s Hospital of Nanjing Medical University. The research related to animals’ use has been complied with all the relevant national regulations and institutional policies for the care and use of animals.

### 2.13 Statistical analysis

All statistical results were expressed as mean ± standard deviation based on three independent experiments. Differences between groups were compared by Student’s *t*-test or one-way analysis of variance. Statistical analysis was performed using GraphPad Prism software version 6.0 (GraphPad, La Jolla, CA, USA). *P* < 0.05 was considered significant.

## 3 Results

### 3.1 The upregulation of circ_0030018 was found in glioma tissues and cells

**Figure 1:**
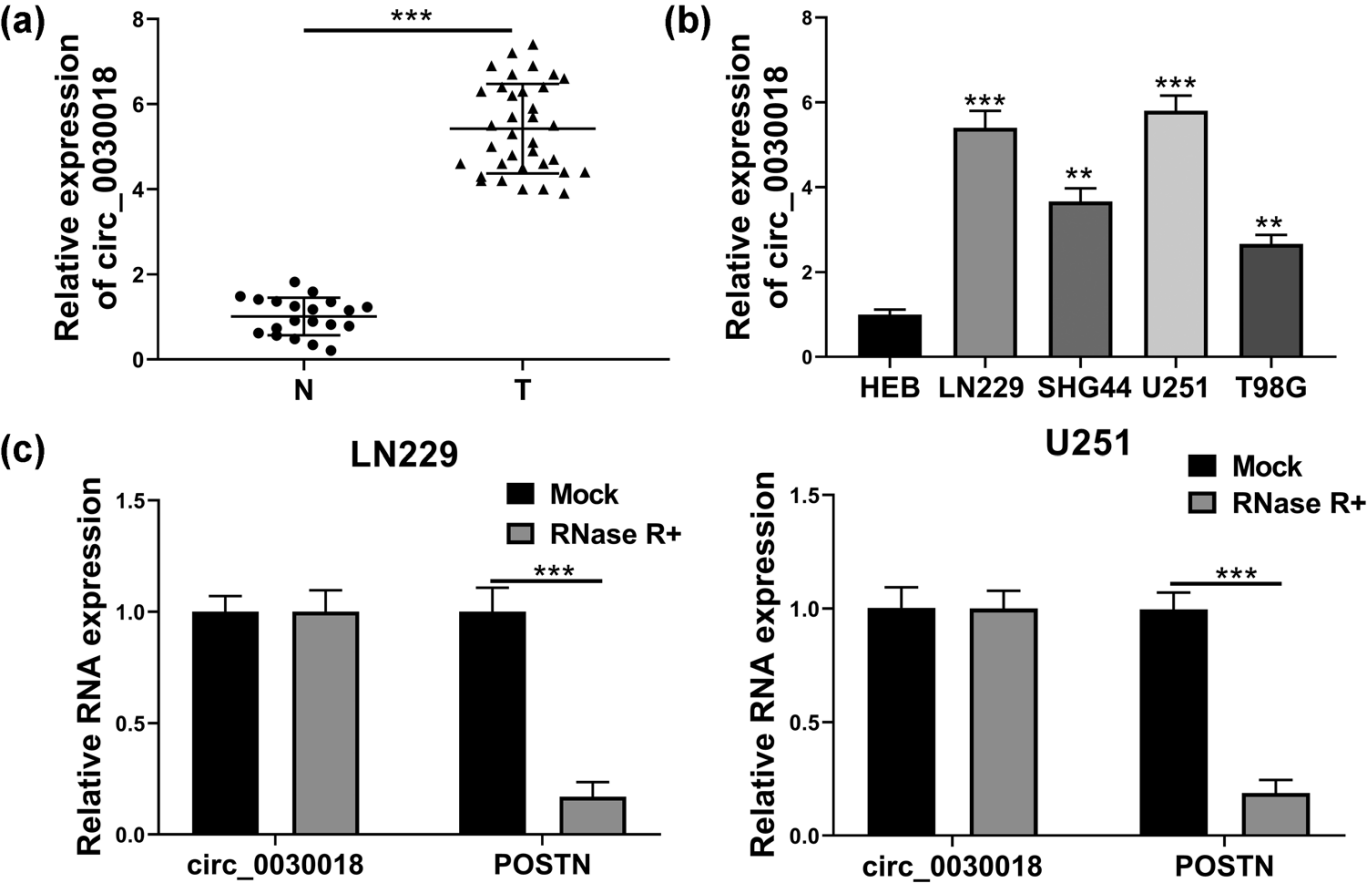
circ_0030018 was upregulated in glioma tissues and cells. (a) The expression of circ_0030018 in glioma tissues (T) and normal brain tissues (N) was detected by qRT-PCR. (b) circ_0030018 expression in HEB cells and glioma cells (LN229, SHG44, U251, and T98G) was assessed by qRT-PCR. (c) RNase R assay was used to evaluate the stability of circ_0030018 and POSTN in LN229 and U251 cells. **P < 0.01, ***P < 0.001.

In glioma tissues and normal brain tissues, the expression of circ_0030018 was found to be highly expressed in glioma tissues markedly (Figure 1a). Further, circ_0030018 also was upregulated in four glioma cells (especially in LN229 and U251 cells) compared with that in HEB cells (Figure 1b). Using RNase R assay, we found that circ_0030018 expression was not affected by RNase R digestion, whereas linear *POSTN* could be degraded by RNase R in LN229 and U251 cells (Figure 1c). Therefore, our data showed that circ_0030018 did have a circular structure, which was more stable than linear RNA.

### 3.2 circ_0030018 knockdown suppressed glioma cell proliferation, migration, invasion, and enhanced apoptosis

**Figure 2:**
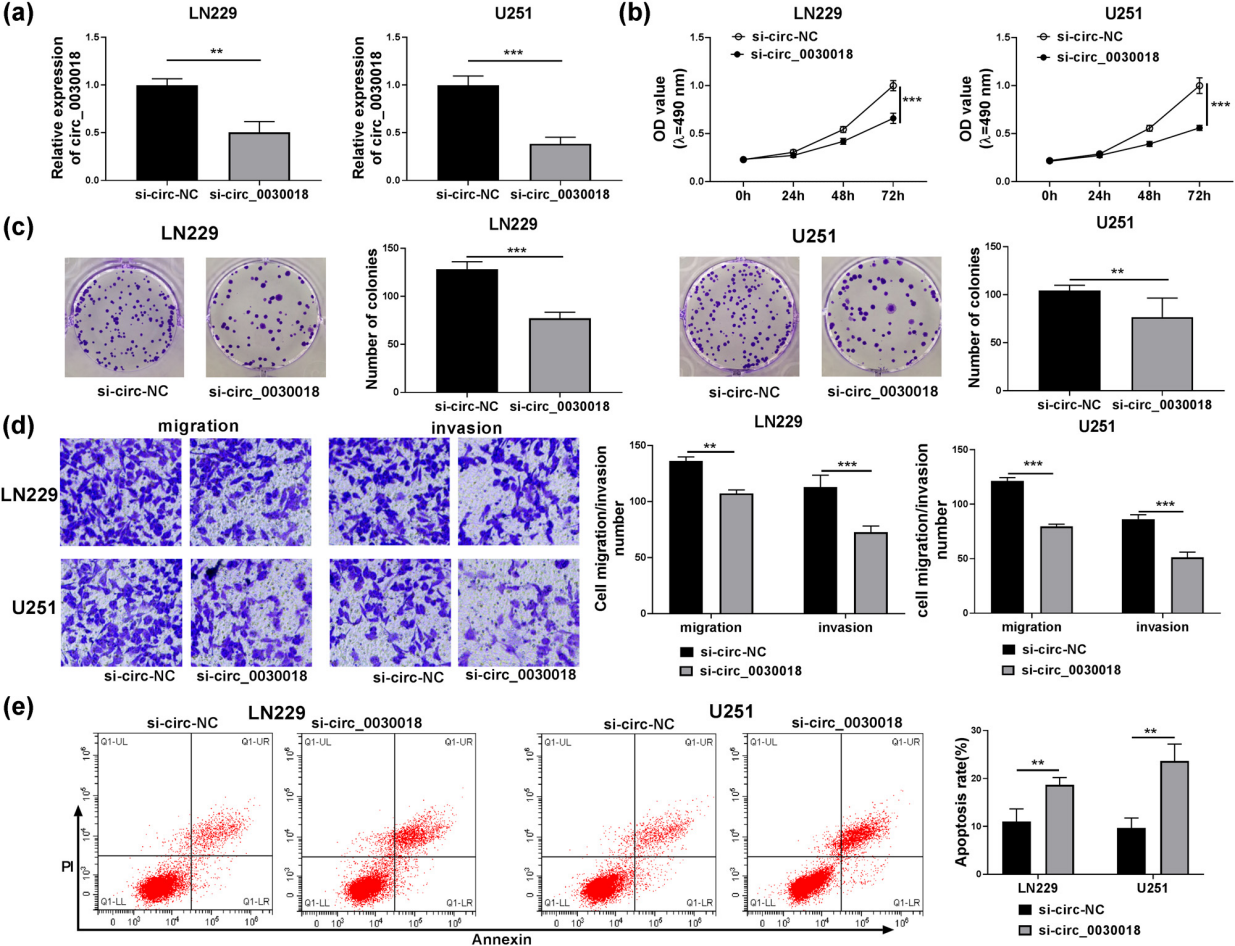
circ_0030018 knockdown negatively regulated glioma cell progression. LN229 and U251 cells were transfected with si-circ-NC or si-circ_0030018. (a) The expression of circ_0030018 was measured by qRT-PCR. (b) The viability of cells was detected by MTT assay. (c) Colony formation assay was used to assess the number of colonies. (d) The migration and invasion of cells were determined by transwell assay. (e) Flow cytometry was performed to test cell apoptosis rate. **P < 0.01, ***P < 0.001.

For illuminating the function of circ_0030018 in glioma, si-circ_0030018 was constructed and transfected into LN229 and U251 cells. As presented in Figure 2a, circ_0030018 expression was indeed reduced in LN229 and U251 cells transfected with si-circ_0030018. In addition, we measured linear POSTN expression and confirmed that circ_0030018 knockdown did not affect the expression of POSTN at the mRNA level and protein level (Figure S1a and b). Then, we evaluated the biological functions of glioma cells. The results suggested that circ_0030018 silencing inhibited the viability and the number of colonies in LN229 and U251 cells (Figure 2b and c). Moreover, the migration and invasion numbers of LN229 and U251 cells also were reduced after circ_0030018 knockdown (Figure 2d). In addition, silenced circ_0030018 also promoted the apoptosis rate of LN229 and U251 cells, as demonstrated by flow cytometry (Figure 2e).

### 3.3 circ_0030018 could act as a sponge of miR-194-5p

**Figure 3:**
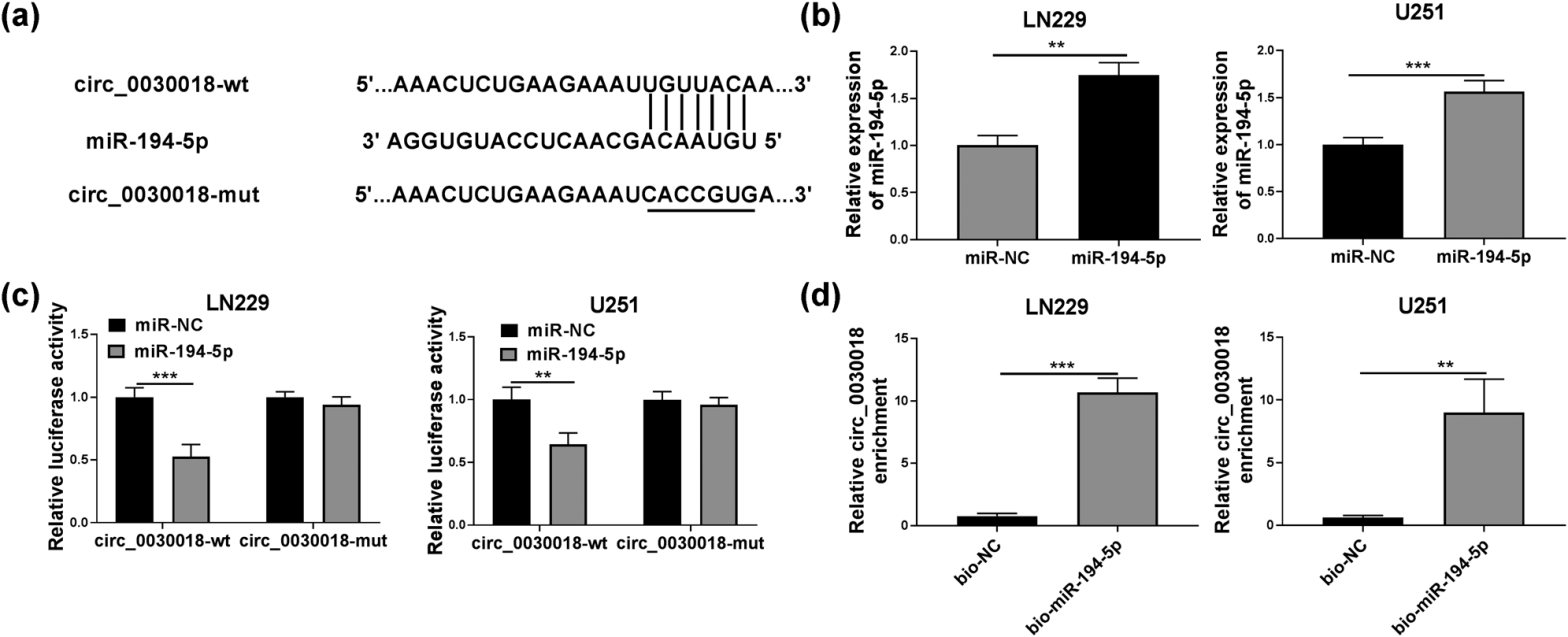
circ_0030018 could act as a sponge of miR-194-5p. (a) The putative binding sites of miR-194-5p in circ_0030018 were presented. (b) The expression of miR-194-5p in LN229 and U251 cells transfected with miR-NC or miR-194-5p was detected by qRT-PCR. Dual-luciferase reporter assay (c) and RNA pull-down assay (d) were used to verify the interaction between circ_0030018 and miR-194-5p. **P < 0.01, ***P < 0.001.

To explore the mechanism of circ_0030018 regulated glioma progression, the circinteractome software was used to predict the targeted miRNA of circ_0030018. As shown in Figure 3a, circ_0030018 was found to have binding sites with miR-194-5p (TGTTACA). Then, miR-194-5p mimic was built and its transfection efficiency was confirmed through detecting miR-194-5p expression in LN229 and U251 cells transfected with miR-194-5p mimic (Figure 3b). By measuring the mRNA and protein expression of POSTN, we found that miR-194-5p overexpression had no effect on POSTN expression (Figure S1c and d), which confirmed that there was no interaction between miR-194-5p and *POSTN*. Using the dual-luciferase reporter assay, we discovered that the luciferase activity of circ_0030018-wt vector could be reduced by miR-194-5p mimic, but that of the circ_0030018-mut vector had not changed (Figure 3c). Also, the enrichment of circ_0030018 was markedly pulled down by the bio-miR-194-5p probe compared to the bio-NC probe (Figure 3d). These results suggested that circ_0030018 could interact with miR-194-5p in glioma.

### 3.4 miR-194-5p inhibited proliferation, metastasis, and increased apoptosis in glioma cells

**Figure 4:**
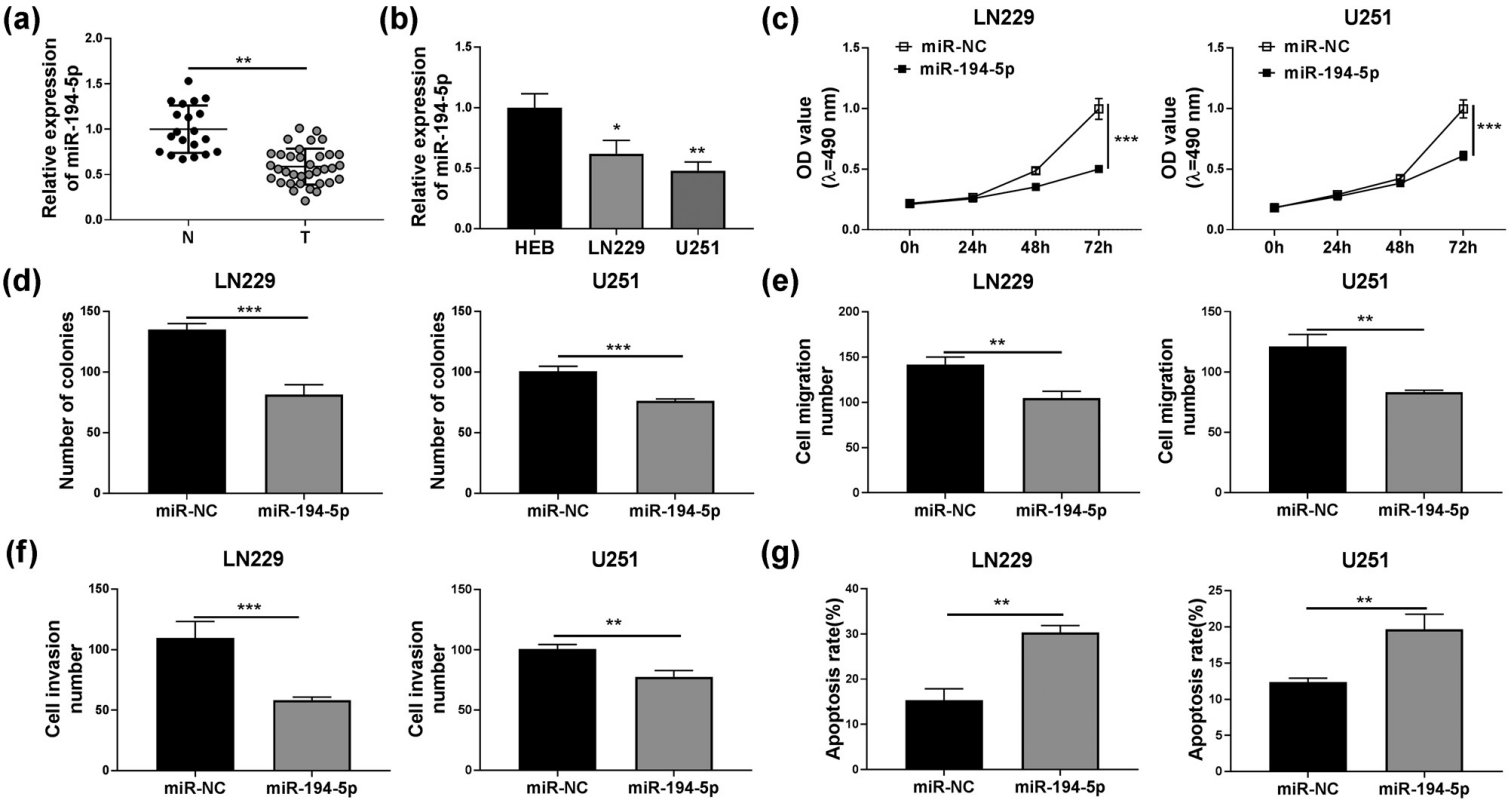
miR-194-5p inhibited glioma cell progression. (a) miR-194-5p expression in glioma tissues (T) and normal brain tissues (N) was examined by qRT-PCR. (b) QRT-PCR was used to detect miR-194-5p expression in HEB cells and glioma cells (LN229 and U251). (c–g) LN229 and U251 cells were transfected with miR-194-5p mimic or miR-NC. (c) MTT assay was used to detect cell viability. (d) The number of colonies was assessed by colony formation assay. (e and f) Transwell assay was used to determine the migration and invasion of cells. (g) The apoptosis rate of cells was measured by flow cytometry. *P < 0.05, **P < 0.01, ***P < 0.001.

Compared to normal brain tissues, miR-194-5p was remarkably downregulated in glioma tissues (Figure 4a). And the expression of miR-194-5p in LN229 and U251 cells was higher than that in HEB cells (Figure 4b). To investigate the role of miR-194-5p in glioma, miR-194-5p mimic was transfected into LN229 and U251 cells. MTT assay and colony formation assay showed that the viability and the colony number of LN229 and U251 cells were obviously suppressed after miR-194-5p overexpression (Figure 4c and d). Besides, overexpressed miR-194-5p also could inhibit the migration and invasion of LN229 and U251 cells (Figure 4e and f). Through the flow cytometry assay, we also observed that cell apoptosis rate was notably increased in LN229 and U251 cells transfected with miR-194-5p mimic (Figure 4g).

### 3.5 circ_0030018 regulated glioma progression via sponging miR-194-5p

**Figure 5:**
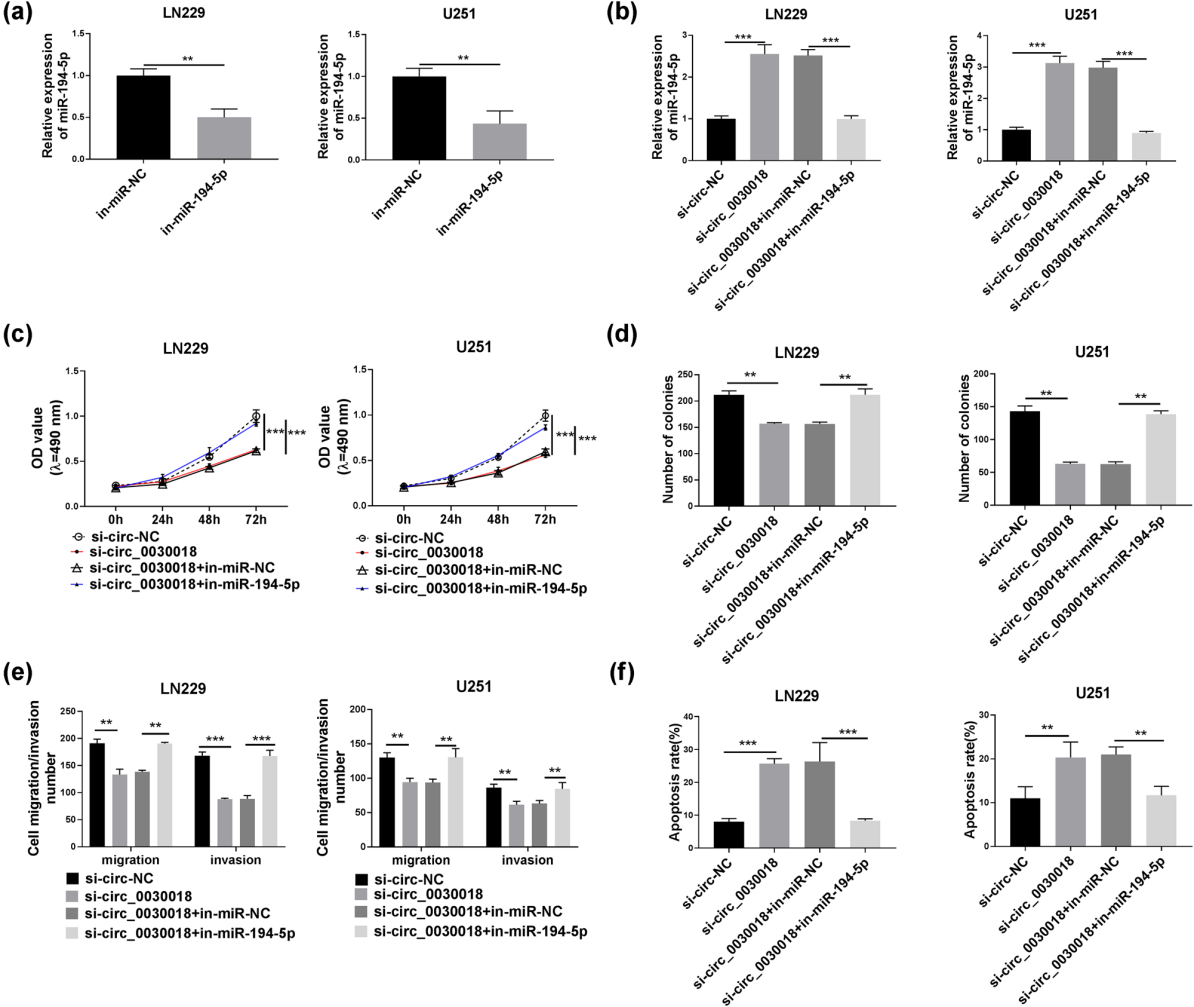
circ_0030018 regulated glioma progression via sponging miR-194-5p. (a) miR-194-5p expression in LN229 and U251 cells transfected with in-miR-NC or in- miR-194-5p was detected by qRT-PCR. (b–f) LN229 and U251 cells were transfected with si-circ-NC, si-circ_0030018, si-circ_0030018 + in-miR-NC, or si-circ_0030018 + in-miR-194-5p. (b) QRT-PCR was used to detect miR-194-5p expression. (c) The viability of LN229 and U251 cells was assessed by MTT assay. (d) Colony formation assay was employed to detect the number of colonies. (e) The migration and invasion of cells were evaluated using transwell assay. (f) The apoptosis of cells was evaluated using flow cytometry. **P < 0.01, ***P < 0.001.

After transfecting with in-miR-194-5p into LN229 and U251 cells, we found that miR-194-5p expression was markedly decreased (Figure 5a). Then, we assessed the biological functions of LN229 and U251 cells. The results of function experiments showed that miR-194-5p inhibitor could promote the colony number, migration number, and invasion number, while reduced the apoptosis of LN229 and U251 cells (Figure S2a–d). To perform further experiments to confirm whether circ_0030018 sponged miR-194-5p to regulate glioma progression, si-circ_0030018 and in-miR-194-5p were co-transfected into LN229 and U251 cells. The expression of miR-194-5p could be enhanced by circ_0030018 silencing, while this effect could be reversed by miR-194-5p inhibitor (Figure 5b). Through measuring the biological functions of LN229 and U251 cells, we discovered that the inhibitory effects of circ_0030018 on the viability, colony number, migration, and invasion of LN229 and U251 cells could be abolished by miR-194-5p inhibitor (Figure 5c–e). Furthermore, miR-194-5p inhibitor also reversed the increasing effect of circ_0030018 knockdown on the apoptosis rate of LN229 and U251 cells (Figure 5f).

### 3.6 *TRIM44* was targeted by miR-194-5p

**Figure 6:**
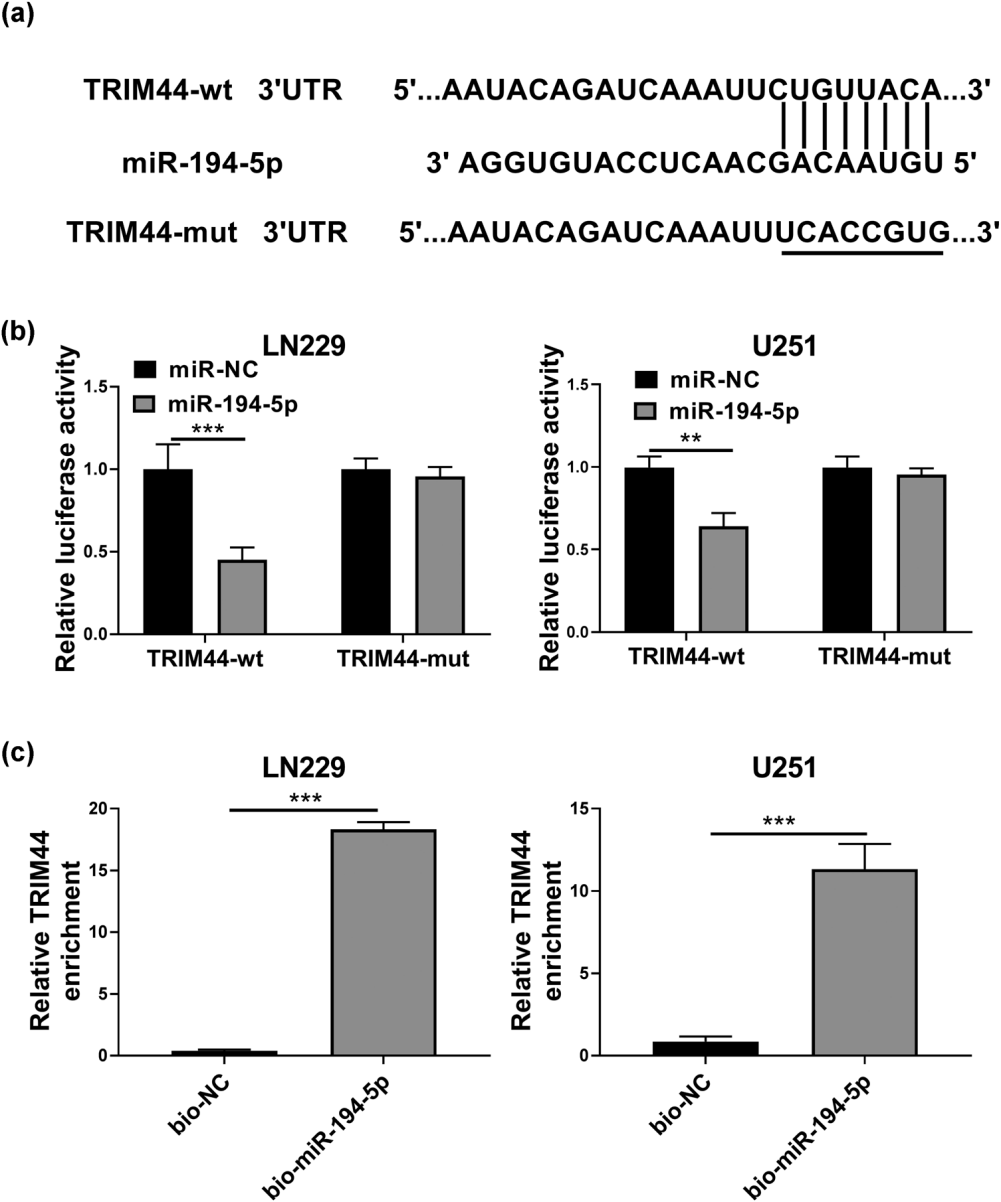
TRIM44 was targeted by miR-194-5p. (a) The putative binding sites between TRIM44 3′UTR and miR-194-5p were shown. The interaction between TRIM44 and miR-194-5p was confirmed by dual-luciferase reporter assay (b) and RNA pull-down assay (c). **P < 0.01, ***P < 0.001.

The Targetscan tool was used to predict the targets of miR-194-5p. The 3′UTR of *TRIM44* was found to bind with miR-194-5p (Figure 6a). The results of dual-luciferase reporter assay showed that miR-194-5p mimic could inhibit the luciferase activity of *TRIM44*-wt vector without affecting that of the *TRIM44*-mut vector in LN229 and U251 cells (Figure 6b). Besides, RNA pull-down assay revealed that compared to the bio-NC probe, *TRIM44* was enriched in the bio-miR-194-5p probe (Figure 6c).

### 3.7 Silenced TRIM44 had an inhibition effect on glioma progression

**Figure 7:**
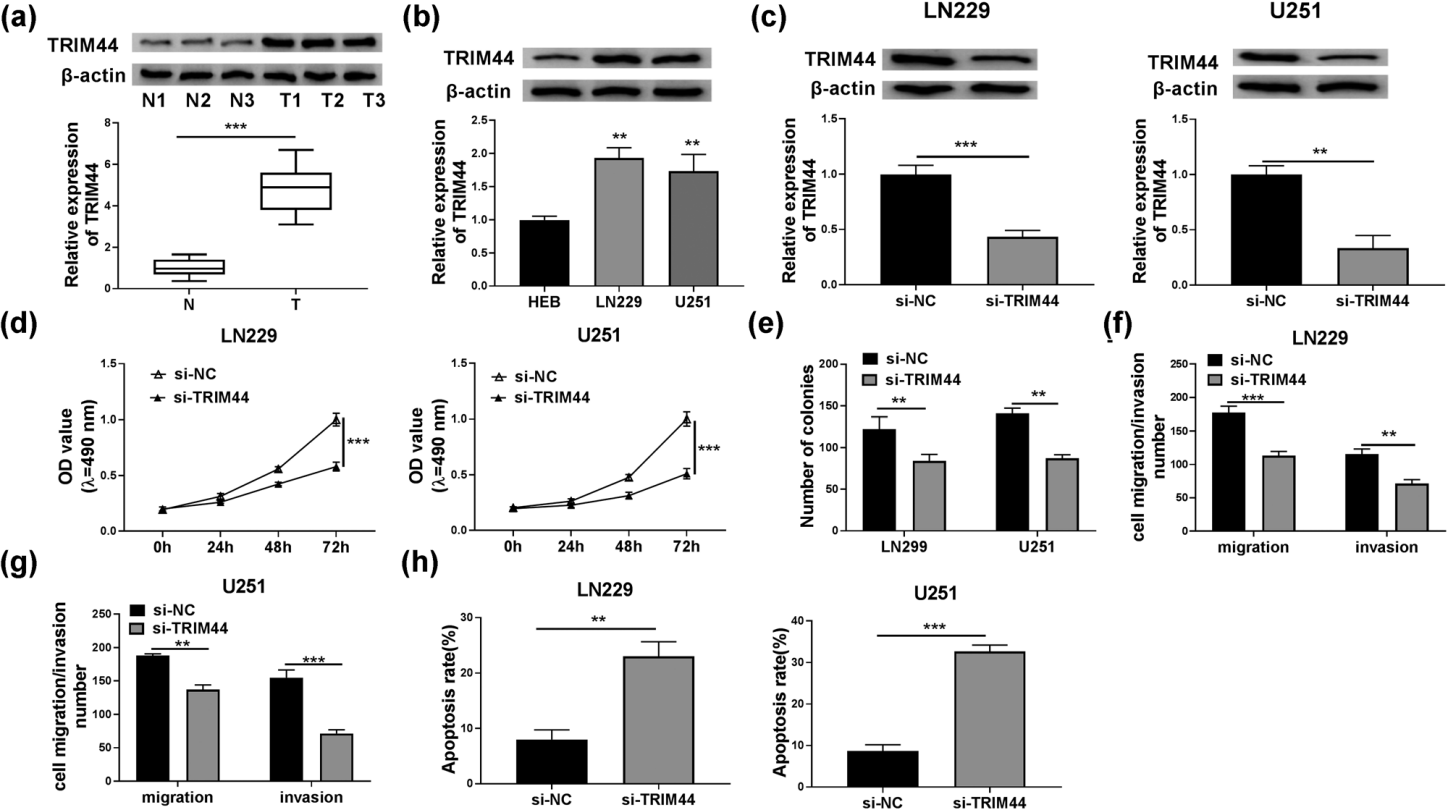
Silenced TRIM44 had an inhibition effect on glioma progression. (a) The protein expression of TRIM44 in glioma tissues (T) and normal brain tissues (N) was measured by WB analysis. (b) WB analysis was used to examine the protein expression of TRIM44 in HEB cells and glioma cells (LN229 and U251). (c–h) LN229 and U251 cells were transfected with si-NC or si-TRIM44. (c) TRIM44 protein expression was detected by WB analysis. (d) Cell viability was determined using MTT assay. (e) The number of colonies was measured by colony formation assay. (f and g) The migration and invasion of cells were tested using transwell assay. (h) Cell apoptosis rate was evaluated by flow cytometry. **P < 0.01, ***P < 0.001.

By detecting TRIM44 protein expression in glioma tissues and cells, we discovered that TRIM44 was obviously overexpressed compared with corresponding controls (Figure 7a and b). To confirm the role of TRIM44 in glioma, si-TRIM44 was used to decrease TRIM44 expression in LN229 and U251 cells (Figure 7c). Function experiments revealed that TRIM44 silencing could hinder the viability, colony numbers, migration, and invasion of LN229 and U251 cells (Figure 7d–g). And the apoptosis of LN229 and U251 cells also could be accelerated by TRIM44 knockdown (Figure 7h).

### 3.8 Overexpressed TRIM44 reversed the regulation of miR-194-5p on glioma progression

**Figure 8:**
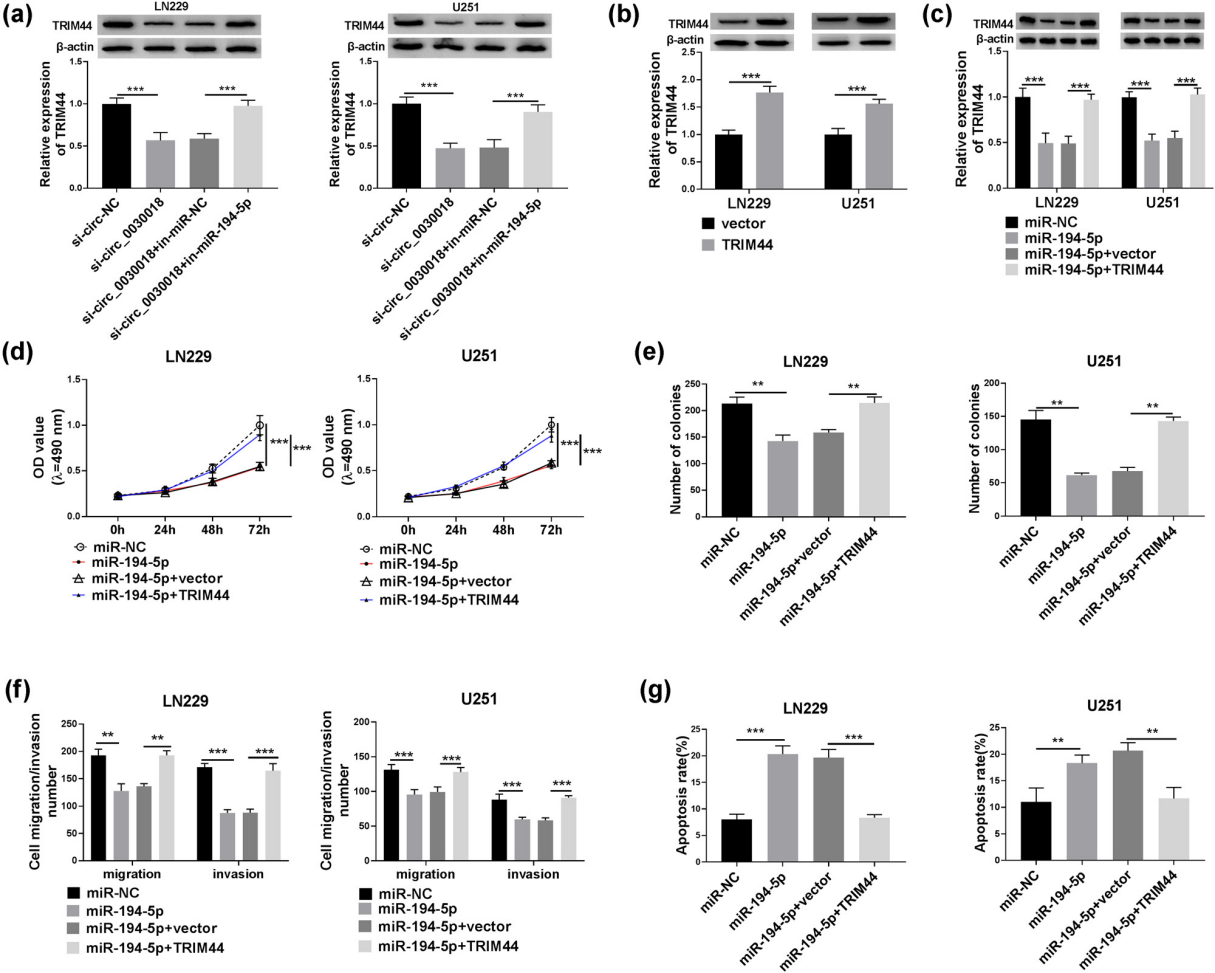
Overexpressed TRIM44 reversed the regulation of miR-194-5p on glioma progression. (a) The protein expression of TRIM44 was assessed by WB analysis in LN229 and U251 cells transfected with si-circ-NC, si-circ_0030018, si-circ_0030018 + in-miR-NC, or si-circ_0030018 + in-miR-194-5p. (b) In LN229 and U251 cells transfected with vector or TRIM44 overexpression vector, the TRIM44 protein expression was detected by WB analysis. (c–g) LN229 and U251 cells were transfected with miR-NC, miR-194-5p, miR-194-5p + vector, or miR-194-5p + TRIM44. (c) TRIM44 protein expression was measured using WB analysis. (d) MTT assay was performed to measure cell viability. (e) The number of colonies was determined using colony formation assay. (f) Transwell assay was employed to test cell migration and invasion. (g) Flow cytometry was used to examine the apoptosis rate of cells. **P < 0.01, ***P < 0.001.

In LN229 and U251 cells transfected with si-circ_0030018 and in-miR-194-5p, we found that circ_0030018 silencing could obviously decrease TRIM44 protein expression, and its effect could be reversed by miR-194-5p inhibitor (Figure 8a). These suggested that circ_0030018 regulated TRIM44 by sponging miR-194-5p. To further confirm that miR-194-5p regulated glioma progression by targeting TRIM44, TRIM44 overexpression vector was constructed and further analysis confirmed that it indeed markedly enhanced TRIM44 protein expression (Figure 8b). Subsequently, miR-194-5p mimic and TRIM44 overexpression vector were co-transfected into LN229 and U251 cells. The TRIM44 protein expression inhibited by miR-194-5p could be reversed by TRIM44 overexpression, indicating that both transfection efficiencies were good (Figure 8c). As exhibited in Figure 8d–f, the suppressive effect of miR-194-5p on the viability, colony number, migration, and invasion of LN229 and U251 cells could be abolished by TRIM44 overexpression. Similarly, upregulation of TRIM44 also could reverse the promotion effect of miR-194-5p on the apoptosis of LN229 and U251 cells (Figure 8g).

### 3.9 Interference of circ_0030018 reduced glioma tumor growth

**Figure 9:**
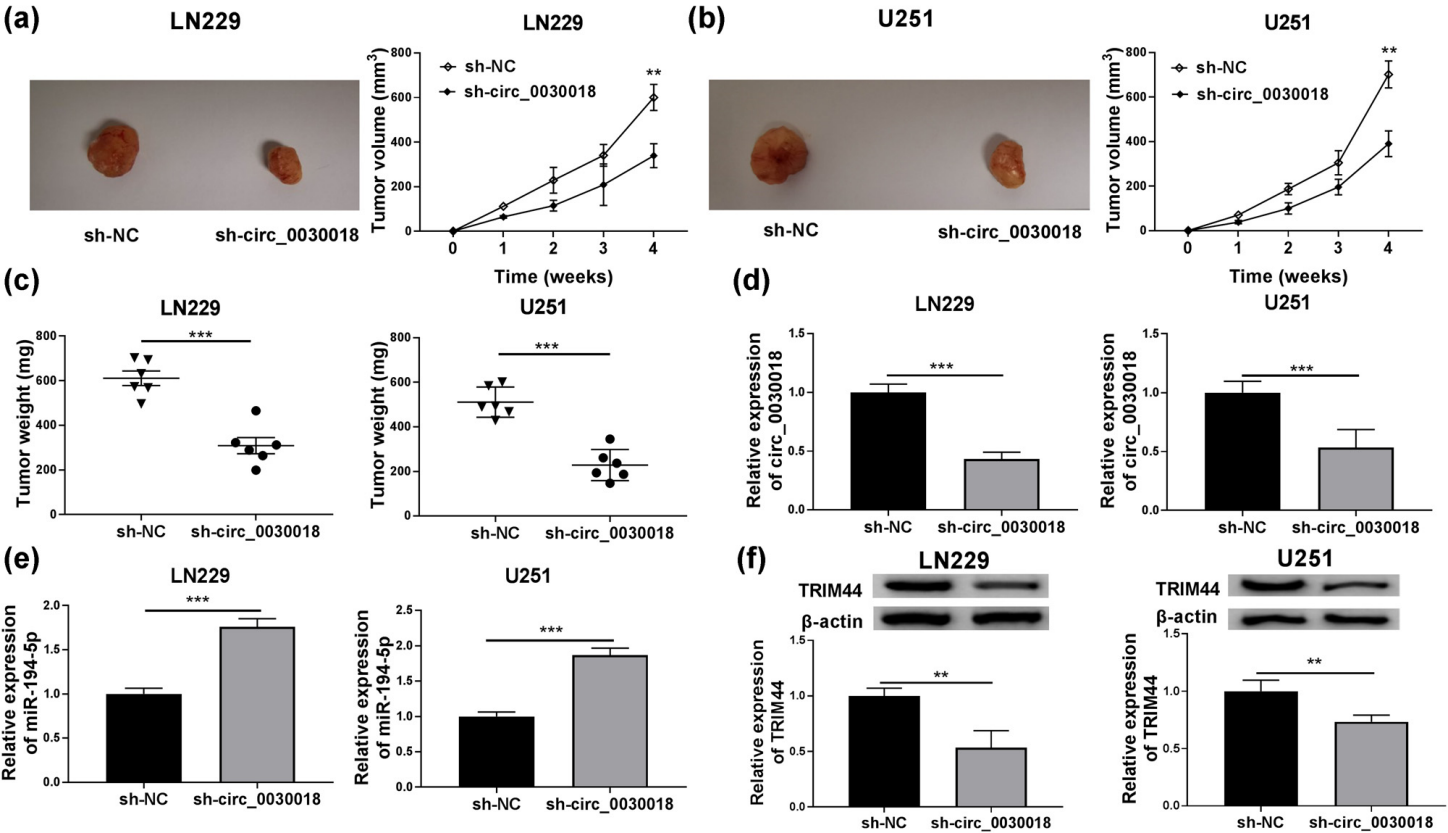
Interference of circ_0030018 reduced glioma tumor growth. LN229 and U251 cells transfected with sh-circ_0030018 or sh-NC were injected into nude mice. (a and b) The tumor volume of each group was detected. (c) The tumor weight was measured after four weeks. (d and e) The expression of circ_0030018 and miR-194-5p in the tumors of mice was measured by qRT-PCR. (f) WB analysis was used to examine the protein expression of TRIM44 in the tumors of mice. **P < 0.01, ***P < 0.001.

To further verify the role of circ_0030018 in glioma, LN229 and U251 cells transfected sh-circ_0030018 were injected into nude mice to perform *in vivo* experiments. After four weeks, we found that the tumor volume of mice in the sh-circ_0030018 group was smaller than that in the control group (Figure 9a and b). The detection results of tumor weight confirmed that compared to the control group, the tumor weight of mice in the sh-circ_0030018 group was remarkably decreased (Figure 9c). In addition, the detection of circ_0030018 expression in tumor tissues confirmed that circ_0030018 expression was indeed inhibited in the sh-circ_0030018 group (Figure 9d). At the same time, we also detected a significant increase in the expression of miR-194-5p and a significant decrease in the protein expression of TRIM44 in tumor tissues of sh-circ_0030018 group (Figure 9e and f).

## 4 Discussion

At present, many circRNAs have been found to regulate glioma progression. For example, circHIPK3 was upregulated in glioma, which promoted glioma cell proliferation and invasion via the miR-654/IGF2BP3 axis [22]. circ_0034642 was found to be overexpressed in glioma, which could sponge miR-1205 to accelerate glioma proliferation and metastasis by regulating BATF3 [23]. On the contrary, circ_0008225 had been discovered to play a tumor suppressor role in glioma, which could inhibit the tumorigenesis of glioma through regulating the miR-8909/ZMYND11 network [24]. Therefore, circRNA is a potential biomarker for glioma treatment and prognosis.

In this, we investigated the role of circ_0030018 in glioma. Our study suggested that circ_0030018 was highly expressed in glioma. Besides, loss-of-function experiments showed that circ_0030018 knockdown negatively regulated glioma cell proliferation, migration, and invasion, while markedly promoted apoptosis. These data suggested that circ_0030018 functioned as a tumor promoter in glioma, which was consistent with the results proposed by Yang et al. [14]. In addition, we also conducted *in vivo* experiments. The new results showed that circ_0030018 silencing could suppress glioma tumor growth, which confirmed that knockdown of circ_0030018 might be an effective way to inhibit glioma malignant progression.

miR-194-5p has been shown to be significantly underexpressed in many tumors. Wang et al. showed that miR-194-5p reduced breast cancer migration and invasion to inhibit cancer progression [25]. In gastric cancer, miR-194-5p was considered to be tumor suppressor to hinder cancer cell proliferation and invasion [26]. Importantly, previous study indicated that miR-194-5p could suppress the tumor growth of glioblastoma multiforme [27,28], and it also had the negative role in glioma malignant progression [29,30]. Here, we found that circ_0030018 could act as miR-194-5p sponge. Similar to the previous studies [29,30], our data confirmed that miR-194-5p was downregulated in glioma and could restrain glioma cell proliferation and metastasis and enhance apoptosis. Further analysis suggested that circ_0030018 knockdown inhibited glioma progression could be reversed by miR-194-5p inhibitor, revealing that circ_0030018 sponged miR-194-5p to regulate glioma progression.

Furthermore, we discovered that *TRIM44* was targeted by miR-194-5p. *TRIM44* is a member of TRIM family. It has been found that TRIM44 is overexpressed in a variety of tumors and is involved in malignant tumor progression, including colorectal cancer [31], esophageal cancer [32], renal cell carcinoma [33], and breast cancer [34]. Li et al. reported that miR-101-3p could target TRIM44 to inhibit glioblastoma proliferation and metastasis [35]. In addition, other studies confirmed that the high TRIM44 expression was closely related to the proliferation and cell cycle progression of glioma [36,37]. Consistent with the previous conclusions, our study suggested that TRIM44 deletion could inhibit glioma progression. The rescue experiments revealed that TRIM44 could reverse the inhibition effect of miR-194-5p on glioma progression, which illuminated that miR-194-5p targeted TRIM44 to participate in the regulation of glioma. Moreover, we also proposed that circ_0030018 negatively regulated TRIM44 by targeting miR-194-5p *in vitro* and *in vivo*.

Collectively, our results indicated that circ_0030018 promoted glioma proliferation and metastasis by regulating the miR-194-5p/*TRIM44* axis. These findings confirmed the important role of circ_0030018 in glioma progression and provided a feasible idea for glioma treatment. In addition, the new mechanism of circ_0030018 also provided reference for the relevant research of circ_0030018.
